# Assessing the implementation of community-based learning in public health: a mixed methods approach

**DOI:** 10.1186/s12909-021-03098-5

**Published:** 2022-01-17

**Authors:** Pierre Leblanc, Pauline Occelli, Jerome Etienne, Gilles Rode, Cyrille Colin

**Affiliations:** 1 Direction Qualité Usagers et Santé Populationnelle (DQUSP), Hospices Civils de Lyon, Lyon, France; 2 Research On Healthcare Performance (RESHAPE), Université Claude Bernard Lyon 1, INSERM U1290, Lyon, France; 3 Pôle de Santé Publique, Hospices Civils de Lyon, Lyon, France; 4 Faculté de Médecine Lyon Est, Université Claude Bernard, Lyon 1, Lyon, France; 5 Service d’Evaluation Economique en Santé Pôle de Santé Publique, Hospices Civils de Lyon, Lyon, France

**Keywords:** Public health, Health promotion, Undergraduate, Professionalism, Community-oriented

## Abstract

**Background:**

The French government has set up a community-based learning programme on health promotion for undergraduate health students to involve them in key public health objectives. At the University of Lyon, students first underwent formal instruction, including e-learning, lectures, and interactive seminars, and then became health educators for school pupils. The main objective of the present study was to assess the process of implementing this programme during the 2018–2019 academic year.

**Methods:**

The satisfaction and perception of medical and midwife students with community-based learning experiences were assessed by a questionnaire, semi-directive interviews, and observations. Replies to the questionnaire were described by median and interquartile range or by proportion. A paired Wilcoxon-Mann–Whitney test was used to compare self-evaluated students’ competence scores before and after the seminars (alpha risk of 5%). Thematic analyses using grounded theory were performed on recorded and transcribed interviews, and on transcribed notes taken during the observations.

**Results:**

Over time the students have evolved from a negative perception of the community-based learning to a positive one. The students were mostly satisfied by interactive seminars that allowed them to gain confidence and competencies in health education. Their involvement in the programme increased their self-esteem. They became more aware of their educative responsibilities regarding public health issues as future professionals.

**Conclusions:**

The students had a positive perception of the implementation of a community-based learning programme in our University, as it appeared a pertinent strategy to raise their awareness of prevention and health education issues.

**Supplementary Information:**

The online version contains supplementary material available at 10.1186/s12909-021-03098-5.

## Background

The promotion of preventive healthcare is insufficient in France and remains poorly organised. There is a lack of coherent coordination between French agencies involved in prevention, and a mismatch exists between needs and funding policy. Furthermore, healthcare providers devote only a small portion of their activity to this field, which is strategic for the population’s health [1]. Prevention in the French healthcare system lags behind levels found in other developed countries such as the United Kingdom, Canada, and Finland [2–4]. The lack of a broad national prevention policy also results in a lack of training in prevention for health students.

In 2018, the French government set up an educational programme on health prevention for undergraduate health students [5]. In France, the designation “undergraduate health student” refers to health students between their A-level degree(s) obtained at the end of high school and their graduation obtained before the beginning of their residency. In the first year of implementation, a total of 47,000 students were included at the national level. This educational programme has two main objectives: (i) introduce future health professionals to the challenges of primary prevention and health promotion and develop skills for integrating these concepts into their future clinical practice; (ii) ensure health education actions, targeting disadvantaged populations. This programme consists of theoretical training within universities followed by practical training in the form of community-based learning on themes for which primary prevention is needed.

In 2018, two themes were selected in the health faculties of the University of Lyon: (i) lifestyle habits and behaviours with topics such as diet, physical activity, sleep, and oral hygiene; (ii) first aid procedures. The target audience were pupils from primary school to college who were located in priority areas defined by a high proportion of individuals from disadvantaged socio-professional categories.

We report herein the assessment of the process of implementing an educational programme on health prevention in the medical and midwife faculties of the University of Lyon during the academic year of 2018–2019.

## Methods

A mixed methodology was used to assess the students’ experience of this new programme: a quantitative approach based on a questionnaire measuring the students’ satisfaction with the formal education given; a qualitative approach combining semi-directive interviews evaluating the students’ perceptions of the entire programme, and observations of participants in schools to assess how they dealt with the practical training aspects of the programme.

### Theoretical framework

The assessment was based on Donald Kirkpatrick’s model [6]. This framework is used to assess an education programme on four levels: the overall degree of satisfaction of the students (level 1); what they have learnt (level 2); what has changed in their working behaviours (level 3); and what is the long-term impact or the results (level 4). In this study we focused on assessing the first three levels of Kirkpatrick’s model. We did not have the necessary hindsight to evaluate the fourth level of Kirkpatrick’s model (long-term impact) on only one year of the programme. Level 4 of the assessment also requires assessing the effect on a different study population: school pupils or the general population, and not only on students.

### Population

Third-year medical students of the two faculties of medicine and fourth-year midwifery students at the University of Lyon who participated in the community-based learning programme during the academic year 2018–2019 were invited to participate in the quantitative and qualitative approaches.

### Intervention

The educational programme on health prevention had six steps. Firstly, the aims of the programme were presented to the students. Secondly, students followed an e-learning training course on prevention and health promotion. Documentary resources on the selected themes (lifestyle habits and first aid procedures) were available on the faculty’s digital platform. Thirdly, a conventional lecture was organised to reiterate the essential points presented in the e-learning component. Afterwards, twelve hours of interactive seminars over two days allowed students to develop their educational attitude and to discover participative tools to be used during their health education sessions in schools. The pedagogical objectives of the seminars are described in Table 1. Then, the students had to prepare and conduct health education actions for pupils in the schools on one of the two themes. Finally, the students had to present a report on their action.

**Table 1:** Median scores of students’ self-evaluation for the seven objectives of the interactive seminars (*N* = 580)

**Objectives of the interactive seminars**^a^	**Before the seminarMedian [IQR]**	**After the seminarMedian [IQR]**	P**
1. Identify the representations of prevention and the determinants of health	6 [5–7]	7 [6–8]	< 0.001
2. Realise the complexity of health behaviours	7 [5–8]	8 [7, 8]	< 0.001
3. Adopt an adequate educational attitude	6 [5–8]	8 [7, 8]	< 0.001
4. Discover animation techniques according to the educational approach chosen	6 [3–8]	8 [7–9]	< 0.001
5. Build and lead a class session	6 [4–8]	8 [7–9]	< 0.001
6. Understanding of group dynamics	6 [5–8]	8 [7, 8]	< 0.001
7. Able to assess his/her prevention action	6 [5–8]	8 [6–8]	< 0.001
Total score	41 [32–52]	54 [49–58]	< 0.001

^a^1 item per objective: 7 items ranked on continuous numerical scale from 0 to 10

^**^paired Wilcoxon-Mann–Whitney test

*IQR*, interquartile range

### Quantitative method

The main objective was to assess the contribution of the seminars on the development of competencies of the students targeted by this part of the programme (level 2 of Kirkpatrick’s model). The secondary objectives were to assess levels 1 and 3 of Kirkpatrick’s model for the seminars. The methodology described here is reported in a STROBE checklist in Additional file 1.

### Qualitative method

The qualitative approach was aimed at assessing the students’ perception at different steps of the programme (levels 1 and 2 of Kirkpatrick’s model), their appropriation of the educational tools, and a possible change of perception regarding prevention and health promotion (level 3). The methodology described here is reported in a COREQ checklist in Additional file 2.

### Data collection

A self-administered questionnaire was developed for the study. It was composed of 24 items exploring the first three levels of Kirkpatrick’s model: (i) the overall degree of satisfaction of the students (8 items); (ii) what the students have learnt (15 items); and (iii) students’ perception of the impact of the seminars on their future professional practise (1 item). Items were rated on a Likert scale with four levels or on a numerical scale ranging from 0 to 10. The questionnaire was available on the faculty’s digital platform and administered to students at the end of the seminars. The questionnaire used for this study is presented in Additional file 3.

A panel of twenty students received semi-directed interviews at two points during the programme: before their action in schools and at the end of the academic year. Two discussion guides were developed with the help of a sociologist specialised in public health research. The first guide was drafted before any analysis and readjusted after three interviews, while the second guide was drafted after the analysis of the questionnaire and the first interview results. Both the guides used for this study are presented in Additional file 4. The students volunteered to participate. They were first recruited by a call launched on social media (publication on the Facebook students year group), and then by word of mouth. During the recruitment care was taken about recruiting students with different profiles, paying attention to gender, age, the theme dispensed in school, whether they had associative experience or not, military and civilian students, whether elected to the faculty council or not, and future planned specialties. All the interviews were conducted, audio-recorded and transcribed by the first author, and took place at locations chosen by each participant in the faculty building (library, classroom or resting areas outside) with no one else present.

The interviews discussed: (i) students’ perception of public health, prevention, and health promotion; (ii) their opinion of the teaching in terms of form and content (*i.e.,* presentation session, e-learning, lecture, interactive seminars, and practical session on the theme of first aid procedures), and a possible change in their opinion between the first and second interviews; (iii) their opinion on the implementation of the programme; (iv) their implication in the programme; (v) students’ perception of the action they had carried out in schools; (vi) the students’ vision of the value of the community-based learning for them and for civil society; and (vii) the perspectives for improving the programme.

We also conducted observations of students during their actions in schools, to assess their behaviours and attitudes. An observation grid was developed in collaboration with the same sociologist researcher as above and presented in Additional file 5. Observations were also all conducted by the first author and the notes were transcribed on *Microsoft Word*. The students were chosen randomly from among those who were not included for the interviews. They were contacted directly via e-mail by the first author to ask for their consent to participate and be observed during their actions in school. The following was noted: (i) a description of the action content; (ii) their physical posture; (iii) their educational attitudes; (iv) the difficulties they encountered; and (v) feedback or evidence of understanding from the class.

All the participants knew the first author was involved as a teacher in the programme and that his personal goals were to improve the quality of the teaching and the organisation of the programme.

### Data analysis

Replies to the items of the questionnaire were described by median and interquartile ranges [IQR], or by proportion (%). The main endpoint was the students’ perception of their competency or knowledge regarding the seven objectives of the seminars. Each objective was measured by one item of the questionnaire. For each student, a score (out of 70) was calculated by summing the responses of the seven items (Table 1). A paired Wilcoxon-Mann–Whitney test was used to compare scores before and after the seminars with an alpha risk of 5%.

The verbatims of interviews were analysed using the grounded theory method, with the first round of open coding and the second round for axial coding. The identification of redundancies was used to identify data saturation. Notes taken during observations were transcribed for thematic analysis using the grounded theory method with only one round of open coding. No specific software was used for coding the data from the interviews and observations, except *Microsoft Word*.

## Results

### Quantitative study

Among the 640 participating students, 580 answered the questionnaire about the interactive seminars (participation rate: 90.6%).

### Main endpoint

According to the students, their competency/knowledge level improved for the seven objectives of the seminar; the median total score showed a significant increase of 13 points (Table 1).

### Satisfaction with the seminars

**Fig. 1:**
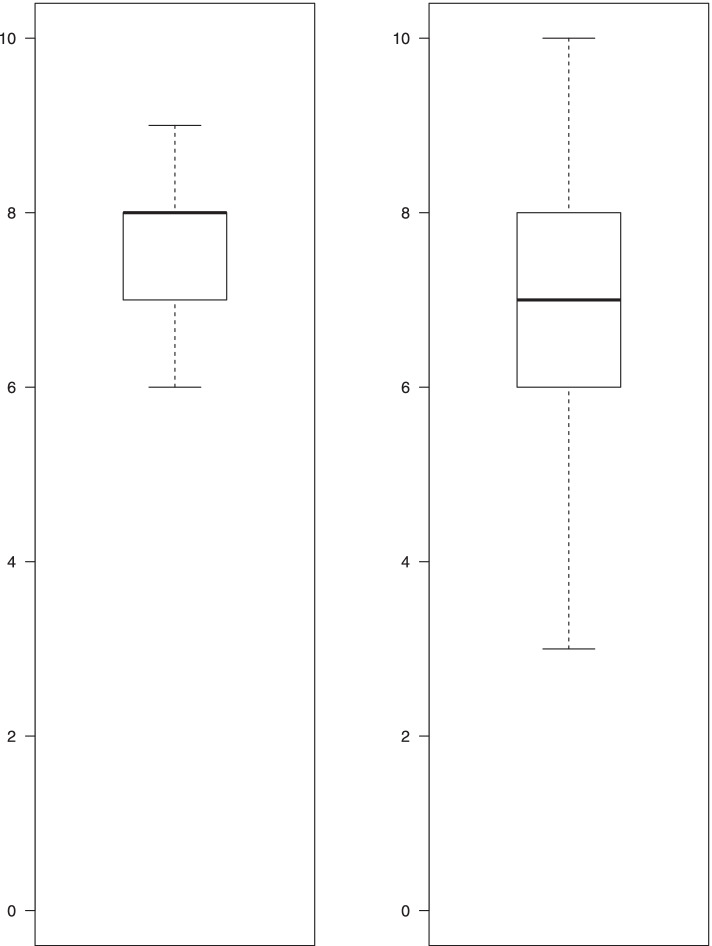
Distribution of students’ ratings on seminars’ quality and their ability to intervene in schools. Left panel: Distribution of students’ rating of the quality of the seminar, using a continuous numerical scale ranging from 0 to 10 (*N* = 579). Right panel: Distribution of students’ self − assessment of their comfort level for carrying out their health education actions in a school environment, using a continuous numerical scale ranging from 0 to 10 (*N* = 535)

The majority of students (82.7%) were quite or very satisfied with the seminars. 89.1% of the students were very or quite satisfied with the facilitation of the seminar, 83.9% of them were very or quite satisfied with the documentation provided, and around 95% were very or quite satisfied with the exchanges between participants, the atmosphere in the group, and the trainers. Three-quarters of the students (75%) considered that the seminars met their expectations totally or partially (Table 2). The median [IQR] attributed to the quality of the seminars was 8 [7, 8] out of 10 (Fig. 1, left panel).

**Table 2:** Satisfaction of students with the seminars, and impact on their professional practice (*N* = 580)

**Level 1 of Kirkpatrick’s model**					
	**Very *N* (%)**	**Quite *N* (%)**	**Not very *N* (%)**	**Not at all *N* (%)**	**No answer *N* (%)**
General satisfaction	145 (25.0)	335 (57.7)	81 (14.0)	19 (3.3)	0 (0)
Satisfaction for:					
1. Seminar facilitation	271 (46.7)	246 (42.4)	36 (6.2)	7 (1.2)	20 (3.5)
2. Exchanges between participants	340 (58.6)	208 (35.9)	11 (1.9)	2 (0.3)	19 (3.3)
3. Documentation provided	182 (31.4)	303 (52.2)	70 (12.1)	7 (1.2)	18 (3.1)
4. Group atmosphere	406 (70.0)	150 (25.9)	7 (1.2)	0 (0.0)	17 (2.9)
5. Atmosphere generated by the pair of trainers	392 (67.6)	159 (27.4)	23 (4.0)	4 (0.7)	2 (0.3)
	Totally N (%)	In part *N* (%)	Weakly *N* (%)	Not at all *N* (%)	No answer *N* (%)
Ability of interactive seminars to meet student expectations	104 (17.9)	331 (57.1)	96 (16.6)	31 (5.3)	18 (3.1)
** Level 3 of Kirkpatrick’s model**					
	Great *N* (%)	Some *N* (%)	Weak *N* (%)	Not at all *N* (%)	No answer *N* (%)
Impact on future practise	55 (9.5)	196 (33.8)	213 (36.7)	77 (13.3)	39 (6.7)

### Students’ competence

The students attributed a median [IQR] score to their feeling of comfort in carrying out their health education action in a school environment of 7 [6–8] out of 10 (Fig. 1, right panel).

### Future professional practice

Half of the students (50.0%) reported that the seminars would have only a weak effect or no effect at all on their future professional practice (Table 2).

### Qualitative study

Half of the 20 students interviewed were women and they were all between 19 and 25 years of age. Half of them intervened on the theme of “lifestyle habits” and the other half on “first aid procedures”. The first series of interviews took place in February 2019 and the second series between May and June 2019. The interviews lasted from 0.5 to 1.5 h in length. Three students were lost to follow-up after the first interview. A selection of verbatims is presented in Table 3. Moreover, six observations of students during their actions in schools were conducted, involving twelve students since they performed actions in pairs. We could not conduct more than six observations as all students performed their actions in school during the same week. Time and human resources of the study were limiting factors.

**Table 3:** Student perceptions deducted from the interviews on community-based service learning in public health

Students’ perception	Narrative illustrations
**1. Benefits of the programme perceived by the students**	
Evolution towards a better perception of the programme	S15-1 ‘I think all the students at first were a bit reticent about that; and now I see everyone is pretty supportive and involved in this thing.’
A better understanding of public health issues	S17-2 ‘I think that is not a bad thing, as in the second and third years, we don’t know much about public health except health economics… which is not really representative of the whole the subject. So, it helps to show that aspect of public health.’
The communication about the objectives of the programme was not clear for students	S15-1 ‘They just told us that we had to do that, but we did not really know why, what it would bring us in the process […] I think that if they had explained to us the whole process of our training, before telling us to do the e-learning, I would certainly have done it more assiduously.’
**2. E-learning as a new way to learn**	
Great benefits from the students’ point of view	S2-1 ‘I think it is not bad, because it allows you to do it at your own pace … at home, quietly. […] so you are sure you can do it.’
Limitations of the e-learning teaching in the programme	S18-1 ‘And on top of that, the fact of not knowing what we answered was more or less right or wrong … It was a bit … not frustrating but […] I said to myself "in fact I don’t know", and after validating (exercises), I still don’t know if what I did was right or not’
**3. Interactive seminars**	
Allow a better understanding of the programme	S2-1 ‘I found it was really much more concrete. Already, this is where they really explained what we were going to do …’
A way to improve student confidence for their actions in schools	S14-1 ‘And I think that reassured me a lot; well for me it reassured me that we already have the ability to do this kind of thing, even if we didn’t necessarily realize it before.’
A lack of scientific knowledge about the theme	S20-1 ‘There was perhaps a lack of training for the notions that we have to deal with. We have training on the form, but not at all on the basic notions’
Participative tools seem useless for students involved in subject of dirst aid procedures actions	S11-1 ‘Because the first part, on how to run a workshop, I think everyone actually should do it. But using the post-its animations, or the moving debate animation, etc.: they seem useless for these in first aid procedures …’
**4. Action in schools**	
Students’ fears	S1-1 ‘And after the worry that will remain until the action, it will be more the question of not losing our means in front of a class.’
Competence in health education	S9-2 ‘Learning how to tell a patient why he should stop smoking or change his behaviour with a different approach: this is more difficult and we don’t necessarily learn that outside of this programme. I think it’s by doing that you actually learn how to do it’
A way to work on communication skills	S7-1 ‘I think it brought me some self-confidence for talking with people and especially in front of a group of people. It was a real training course. And to communicate and interact with our colleagues too.’
Developing human skills, including empathy	S18-2 ‘It is a way of being a bit of a teacher, yes, but also of remaining in our role as a doctor; in fact, not only to provide concrete knowledge, but also to relate to the lives of patients.’ S5-1 ‘It pushed me to see things from other people’s points of view […] and then I learned that a lot of people think differently from me […] it forces me to question everything I know and pay attention to what people want.’ S6-2 ‘I want to have contact with humans, and not beds… and have a more global approach with people.’
Other kinds of skills gained or improved	S2-1 ‘I think it gave me the ability to work in a group, it was nice to work with other students […] We had to organize everything, to work together and communicate; and it seems important to me that we learn all of that.’ S4-2 ‘We met to discuss and decided what activities we were going to use. It was a bit harsh to agree on them and on the subjects or questions we wanted to address […] We obviously didn’t have the same wishes at the start, but step by step we learned how to deal with that and managed to all get together and agree.’
Students’ empowerment	S12-1 ‘And the fact of carrying out these small public health actions, I think that makes sense in our curriculum. It is even empowering in fact.’
**5. Future for the programme**	
Common prevention culture with other health sectors of the University	S12-1 ‘It would be a great opportunity to meet colleagues from other health sectors. So, whether they might be a pharmacist, or a nurse or a dentist, we wouldn’t have the same sensitivities or perspectives on the subjects we deal with, so it would have been an added value for us to be able to exchange in this context.’

The students initially reacted negatively towards the new compulsory teaching programme. However, their attitudes gradually changed during the programme. By the end of the programme, the majority agreed that going into schools themselves to teach school pupils about public health issues was useful. Most of the students interviewed realised the value of being trained in preventative healthcare and gaining a better understanding of key public health issues. They also thought the programme was an opportunity to gain experience in project management, apply principles of health education and prevention, and develop skills in teaching, oral communication, and group work. However, they felt the objectives of the community-based learning were not defined clearly enough, especially regarding the different elements of the programme. The students offered to help clarify these objectives for the following year.

Teaching by e-learning was seen as innovative. This mode of learning allowed students to work from home without a rigid schedule, and thus they were able to organise their work at their own pace. It offered them more time to grasp the theoretical basis of their training before discussing it during lectures and interactive seminars. On the other hand, some students were uncomfortable with the digital format or not motivated by watching videos instead of a real teacher live. The value of written exercises in e-learning was not always understood, with some students preferring more supervision and follow-up by teachers.

Most of the students indicated that they enjoyed the two days of interactive seminars on educational attitudes, which encouraged them to be more participative and gain a better understanding of the purpose of their work in schools. During the seminars they felt empowered to speak to school pupils and gained confidence in doing so. By the end of the two days, the motivation of even the most sceptical students had increased. The opinions of the students appeared divided between those who wished to improve their communication and teaching skills at this stage of their training, and others who felt they needed more medical or scientific knowledge about public health themes. The students were interested in the health education tools presented during the interactive seminars, except for those on first aid procedures, a theme which they felt could be improved.

How the students were received by schools varied from disinterest to enthusiasm, with school nurses playing an important facilitating role. Most of the students described being nervous about having to teach in schools, were lacking in self-confidence, and anxious about interacting with pupils from disadvantaged social backgrounds. They found they had to adapt their language on health issues to suit this unfamiliar audience, some of whom were neither attentive nor interested. The students gained a better understanding of social inequalities in health, health determinants, and health literacy, and their empathy for people affected by these issues increased. They found the teaching experience fulfilling, a feeling reinforced by the positive reaction of school officials. The observations of students showed they understood the principles of participative education, and how to use educational tools appropriately.

The students made various suggestions about how to improve the quality of the programme for future years. Some of them proposed to include complex subjects such as sex education and addiction, for which they felt more training was needed, whilst others would have preferred the option to choose their subject. Some students advocated for better distribution of the time spent on different courses in the academic year to avoid long stretches without teaching followed by periods of intense activity. They also saw working with students from other university health sectors as beneficial for them or actions in schools, and also for developing a shared culture on health.

## Discussion

In this report we have evaluated the implementation of a public health community-based learning programme at the University of Lyon. In this programme medical students were taught the basic concepts of health education and promotion, and then asked to share this knowledge with school pupils. The perception of the students was assessed before and after the learning experience through quantitative and qualitative approaches, and the observations were conducted of the participants in schools. The results indicated that the students were satisfied with teaching by interactive seminars, which were considered to be more concrete than other forms of instruction. Thus, they gained confidence in their teaching capacities. Their perception of the public health programme evolved during the year, from a negative one – due to the new and compulsory nature of the concept – to a positive one after their action in schools. Following their involvement in community-based learning, the students began to broaden their understanding of their role as future physicians in promoting and educating people about public health issues, and gained insight into social inequalities in health, health determinants, and health literacy. The students therefore developed professional skills including knowledge, project management, communication, health education, and empathy. The observation of students during their action showed that they had an adequate understanding of public health issues.

This study had certain limitations. For the quantitative approach based on a questionnaire, the most evident limitation was the possible information bias resulting from the assessment of student skills before and after the seminars that were performed at the same time. This bias might have led to overestimating the impact of the seminars. In addition, the students’ ratings could be subjective and student satisfaction could have been influenced by parameters with no relation to the quality of teaching, such as the friendly nature of the teacher or the students’ interest in the subject. This point is still being debated by medical education researchers [7] and could be a limitation in assessing the training, but we do think students are capable of assessing their own progress. Another limitation of the quantitative approach is that the questionnaire was not validated by any pilot study. Concerning the qualitative approach, the limited number of students interviewed led to data saturation; also they may not have been representative of the group for the entire year. The recruitment methodology based on a call on social media probably comprised a selection bias in the inclusion of students for interviews. We sought to minimize this bias by recruiting students with different characteristics. The low number of observations was also a limitation in the qualitative approach, and we could not be sure about data saturation regarding this point. Another limitation in the qualitative approach was that the interviews and observations were conducted and analysed by a single person. There are also strengths that are of note, for instance, there was a high response rate for the quantitative approach (90.6%), which ensured representative answers [8]. Furthermore, the interviews confirmed the high level of satisfaction of the students with the seminars, and the observations demonstrated that the students acquired adequate health education skills. Methodological triangulation was also a strength of our study, whereby qualitative results were confirmed by quantitative data.

The delivery of undergraduate medical education in public health is a worldwide challenge, and diverse designs for the organisation of curriculums to ensure effective public health education have been reported [9–11]. The integration of national public health problems in the training of medical students at the University of Lyon is occurring very gradually: students receive theoretical training on health education only in the third year of their medical studies and in the fourth year of midwifery studies. However, the training programme for medical students in public health issues is much more complete in many other countries. For instance, at the Ege University medical school in Turkey, the first three years of training include 100 h of lectures, 18 sessions of integrated case discussions, and 83 h per student of group activities related to public health. The latter is composed of problem-solving sessions that include the critical appraisal of health data, social determinants of health, and health promotion [12]. In addition, students in their second-year work in primary health centres under the supervision of trained general practitioners for three half-day periods, and third-year students visit the same centres for seven days [12].

In the US, medical schools have experimented with community-based learning as an additional component of medical education for students [13]. For example, in Arkansas a community-based learning experiment was attempted with a vulnerable population (the homeless) for student nurses, the aim of which was to acquire favourable advocacy for this group, to change the nurses’ initial attitudes and perception, and ultimately improve the health of disadvantaged populations [14]. These experiments allowed students to use their academic knowledge and skills in a specific social context [15] and led to an improvement of students’ academic results, a decrease in the students’ fear of populations they did not know, an increase in empathy, and it also had a positive effect on leadership skills [13, 14, 16]. These outcomes are in agreement with the results presented herein.

The UK has taken this step further since, as of 2018, newly qualified physicians have been trained to apply the principles of sustainable healthcare to medical practice [17]. This focuses on the improvement of health and better delivery of healthcare, rather than late intervention in diseases, resulting in benefits to patients and to the environment on which human health depends. This approach emphasises that future physicians should not only integrate prevention and health promotion in their public health roles, but also the dimension of sustainable development [18]. This goes beyond the French programme described here but could be of interest in the future.

## Conclusions

The assessment of the implementation of this new programme confirmed that community-based learning is a good way to increase students’ awareness of prevention and public health issues, and it also highlighted certain areas that require improvement. Our results are consistent with the assessment of the same programme conducted in Paris [19]. The most important result is the integration of students from all healthcare sectors (physicians, pharmacists, midwifes, nurses, physiotherapists, etc.) but this remained a challenge to overcome as each of these professions has its own academic organisation. The development of inter-professional competence is, however, a clearly defined objective and community-based learning is an appropriate means of achieving it. In addition, the length of community-based learning at present is limited to only a few days during a single academic year but should be extended to the entire duration of training so that French students can acquire a deep and lasting awareness of public health issues for better assimilation in their professional practices.

New paths of research should be followed such as including the students from other health sectors participating in community-based learning. They should also involve other stakeholders (university teachers, school teachers and administration, etc.) in the assessment. The long-term effects on the beneficiaries of actions or on students’ practises should be assessed by longer-term studies corresponding to the fourth level of Kirkpatrick’s model. As it is a national programme, a larger study should be discussed to assess the impact on the general population of the implementation of the community-based learning in health faculties.

## Supplementary Information

**Additional file 1.** STROBE checklist for observational studies.

**Additional file 2.** COREQ checklist for qualitative studies.

**Additional file 3.** Questionnaire used for the qualitative approach translated into English. This questionnaire was developed for the purpose of this study and has not been validated by any pilot study.

**Additional file 4.** Interview guides developed and used for the qualitative approach of the study.

**Additional file 5.** Observation grid developed and used for the qualitative approach of the study.

## Data Availability

The datasets used and analysed during the current study are available from the corresponding author on reasonable request.

## Abbreviations

DQUSP: Direction Qualité, Usagers et Santé des Populations
RESHAPE: Research on Healthcare Performance
IQR: Interquartile range
