# Supplementary material for: Assessing the implementation of community-based learning in public health: a mixed methods approach
Source: BMC Med Educ. 2022 Jan 17;22:40. doi: 10.1186/s12909-021-03098-5 (PMC8764809; doi:10.1186/s12909-021-03098-5)
Supplement: Supplementary file 3 — Additional file 3. Questionnaire used for the qualitative approach translated into English. This questionnaire was developed for the purpose of this study and has not been validated by any pilot study. [file 12909_2021_3098_MOESM3_ESM.docx]

<Supplementary file 3: Satisfaction questionnaire used for seminar assessment (translated into English)

1. **Globally, are you satisfied with the seminar you just participated in?**

🞏 Very satisfied 🞏 Quite satisfied 🞏 Not very satisfied 🞏 Not at all satisfied

1. **On a scale between 0 to 10, how do you rate the quality of this seminar?**

0= very low quality / 10= very high quality

0-1-2-3-4-5-6-7-8-9-10

1. **Could you assess this seminar in terms of the following dimensions?**

|  | Very satisfied | Quite satisfied | Not very satisfied | Not at all satisfied |
| --- | --- | --- | --- | --- |
| Seminar facilitation | □ | □ | □ | □ |
| Exchanges between participants | □ | □ | □ | □ |
| Documentation provided | □ | □ | □ | □ |
| Group atmosphere | □ | □ | □ | □ |
| Atmosphere generated by the pair of trainers/teachers | □ | □ | □ | □ |

1. **For each seminar’s objective, estimate your skill or knowledge level:**

0=not at all capable / 10= totally/quite capable.

|  | Before the seminar | After the seminar |
| --- | --- | --- |
| Identify the representations of prevention and the derminants of health. | 0-1-2-3-4-5-6-7-8-9-10 | 0-1-2-3-4-5-6-7-8-9-10 |
| Realise the complexity of health behaviours. | 0-1-2-3-4-5-6-7-8-9-10 | 0-1-2-3-4-5-6-7-8-9-10 |
| Adopt an adequate educational attitude | 0-1-2-3-4-5-6-7-8-9-10 | 0-1-2-3-4-5-6-7-8-9-10 |
| Discover animation techniques according to the educational approach chosen. | 0-1-2-3-4-5-6-7-8-9-10 | 0-1-2-3-4-5-6-7-8-9-10 |
| Build and lead a class session. | 0-1-2-3-4-5-6-7-8-9-10 | 0-1-2-3-4-5-6-7-8-9-10 |
| Understanding of group dynamics. | 0-1-2-3-4-5-6-7-8-9-10 | 0-1-2-3-4-5-6-7-8-9-10 |
| Able to assess his/her prevention action. | 0-1-2-3-4-5-6-7-8-9-10 | 0-1-2-3-4-5-6-7-8-9-10 |

1. **Did this seminar meet your expectations ?**

🞏 Totally 🞏 In part 🞏 Weakly 🞏 Not at all

1. **Did this seminar allow you to feel comfortable to carry out your health education actions in a school environment, as expected in community-based learning?**

0=not at all capable / 10= totally/quite capable.

0-1-2-3-4-5-6-7-8-9-10

1. **In your opinion, will this seminar have an effect on your professional practice?**

| □ Considerably | □ Somewhat | □ Weak | □ Not at all |
| --- | --- | --- | --- |

1. **General comments you want to share**

-------------------------------------------------------------------------------------------------------------------------------------------------------------------------------------------------------------------------------------------------------------------------------------------------------------------------------

**Thank you for your participation!**
