# Supplementary material for: Assessing the implementation of community-based learning in public health: a mixed methods approach
Source: BMC Med Educ. 2022 Jan 17;22:40. doi: 10.1186/s12909-021-03098-5 (PMC8764809; doi:10.1186/s12909-021-03098-5)
Supplement: Supplementary file 4 — Additional file 4. Interview guides developed and used for the qualitative approach of the study. [file 12909_2021_3098_MOESM4_ESM.docx]

Supplementary file 4: Interview discussion guides

**Guide number 1**

**Student characteristics:**

- Age:
- Gender:
- Theme of action in school:
- Planned specialties:
- Other characteristics?

| **Objectives** | **Questions** | | **Stimulus questions (if necessary)** |
| --- | --- | --- | --- |
| Public Health and Health Promotion Perception | | | |
| Understand the student’s relationship and perceptions about Public Health specialties and the field of Prevention and Health Promotion.  Identify the student profiles | Have you ever had to deal with public health issues?  What does prevention and health promotion mean to you?  What does health education mean to you?  Have you ever taken part in a prevention or health education action? | | What does Public Health mean to you?  Do you have one or more concrete examples of actions in these fields?  Any example?  What was it? What led you to do it? Why? |
| E-learning form of teaching | | | |
| Discuss the consistency of the choice of the form of teaching by e-learning followed by interactive face-to-face sessions (IFFS). | What do you think of e-learning to start teaching and training in the program?  Did you find the e-learning format adapted to the content and subjects covered?  What do you think of the interactive face-to-face session that followed?  Would you have preferred one or another form of teaching instead of or in addition to e-learning? | | Explain or argue your answer with yes or no. Examples? What did you think of the duration of the training?  Which ones? What forms would you have liked to have had? |
| E-learning content | | | |
| Understand what emerged and was learnt from the e-learning teaching.  Discuss the student perception of the content. | The goal is not to evaluate your knowledge, but what do you remember of the e-learning teaching?  Have you been particularly interested in one or more e-learning courses?  From another angle, what are the course you did not like or you found more difficult to understand?  Do you think that certain concepts discussed during the e-learning were not developed enough?  On the contrary, do you think certain concepts were over- developed for what you needed?  Do you have anything to say about the interactive face-to-face session?  Did you have other expectations about the content of these courses? | | What was important for you in the e-learning?  Did anything in it seem pointless to you?  Which ones and why?  (if necessary using the summary document of the e-learning course titles). |
| Contribution of the Interactive Seminars | | | |
| Understand student perception and satisfaction of these interactive seminars.  Understand the contribution of the interactive seminars to previous courses by e-learning. | | Did the form of these two days seminars seem appropriate to you?  How did these two days of seminars go?  What were your expectations for these seminars?  What did these seminars bring you?  How did the previous courses prepare you for these seminars? | What did you think of them?  Did they find answers?  Did you miss something in previous courses that could have helped you to better understand the seminars? |
| Student projection in community-based action in school | | | |
| Study how the student perceives his/her role in community-based action, and how is he/she planning the next step(s)  Discuss the general opinion of the student about the community-based service-learning program. | How do you feel about going to school and carry out your action?  How do you plan it?  Do you have experience in public presentation for educational purposes (monitoring, tutoring, classes, etc.)?  What do you think about the community-based service-learning program?  What do you think about its implementation in our university in Lyon? | | Excitement? Impatience? Any fear? Apprehension? Motivation? Other?  What do you think will happen?  Do you think that experience will help you?  Do you think that you are missing some experience to carry out your action?  Is it a good idea for the curriculum? |

**Guide number 2**

| **Objectives** | **Questions** | **Stimulus questions (if necessary)** |
| --- | --- | --- |
| Feedback on action at school | | |
| Understand the overall feeling of students regarding their action.  Discuss the organization of the students.    Determine what knowledge and skills were used by the students for their action.  Determine what students may have found difficult in performing their action.  Discuss the perspective considered by the students after their action. | How did your action go in the school?  What do you think about your action? How did you perceive it?  How did you prepare your action?  Can you describe what you and your group did?  What do you think about it?  How did you define the subject of your action? And the content?  What did you use to prepare and carry out your action?  What difficulties did you encounter during your action? How did you react to it/them?  How did you feel during your action?  How did you feel after your action?  What experience will you remember of your action in the school?  Did the action bring you something? | Positive and/or negative: What? Why?  What classes? What subject was discussed?  How was it at school? With your contact there?  How was it with the other students of your group?  Was it a point of negotiation with the schoolboard or your contact?  What weight did you have in the decisions?  Can you describe what you performed there?  How did the pupils behave? What was the relationship with the teachers or your contact in the school?  Do you think you had a problem?  Did you have any issues in carrying out your action?  And now?  Did you learn something?  Do you understand anything better?  Any personal stuff maybe? |
| Step back from the courses of the program | | |
| Discuss the contribution of the courses to the preparation and execution of the action.  Question the student if he/she changed their mind on the courses since the first interview.  Understand students’ learning needs. | Were the courses of the e-learning session useful?  Were the seminars useful?  Have the courses become clearer now?  Did the organization of e-learning first and then of the seminars seem relevant to you?  How do you see the place of the e-learning courses now?  How would you organize the courses?  In your opinion, could the e-learning and seminars be improved?  What do you think of some students’ suggestions to add more teaching on scientific knowledge?  Do you agree with the other students’ suggestions that faculty teachers should define the entire content of your action?  Can you now identify what you need in the program courses to carry out your action?  What would you like to teach in the program?  In your opinion, what should be added to the course program? | Why? What did you use?  Did you use the tools presented during the seminars?  What educational attitude?  What do you think of them now?  It was useful or not? Why?  Do you think differently now?  Is it better to focus on this knowledge or on teaching skills during this program?  Is the knowledge acquired in other courses sufficient?  If other courses should be added, what would be your suggestions?  Do you think it is better for you to receive some sort of validated content? Or is it better for you to organize something on your own?  Do you think that validated content would be suitable for your action?  Is something missing in the program?  If you had to define the educational program, what would you propose? |
| Global perception of the community-based learning program | | |
| Students’ opinion in the program  Outlook for improvements in the program  Discuss public health policy | Now that the program is finished, what do you think of it?  What did the program bring you?  What would be the elements you would like to keep in the program?  How do you imagine the use of the knowledge acquired in the program in your future practise?  How do you see your role in prevention with your patients or the general public?  In your opinion, what should be improved in the program here in Lyon?  Public Health, especially prevention, is becoming more important in the curriculum: what do you think about it?  For you, what is the role of medical doctors and healthcare workers in prevention?  Does the community-based service learning help students be more aware of promoting prevention and health? | The general principle?  How is it used?  Do you think this program gives the students other benefits?  Are other elements important to you?  Could what you learned be useful to you after university?  What do you think you would like about it?  Would you like new themes?  What do you think of mixing students from different health disciplines?  Do we need more teaching time? More time for conducting the action?  What do you think of the way we assessed your work at the end of the program?  What can we expect from you? |
