# Supplementary material for: Assessing the implementation of community-based learning in public health: a mixed methods approach
Source: BMC Med Educ. 2022 Jan 17;22:40. doi: 10.1186/s12909-021-03098-5 (PMC8764809; doi:10.1186/s12909-021-03098-5)
Supplement: Supplementary file 5 — Additional file 5. Observation grid developed and used for the qualitative approach of the study. [file 12909_2021_3098_MOESM5_ESM.docx]

Supplementary material 5: Observation grid (translated into English)

| Information on the content of student actions  What knowledge is used?  Is the knowledge used understood well by the students?  Do the students use repetition?  Do they use personal elements? | Other notes/ideas |
| --- | --- |
|  |  |
| Physical Posture  Body control and gestures? What position?  Use of space in the classroom?  How do they use the position of pupils in the classroom? |  |
|  |  |
| Educational attitude  Informative?  Injunctive, directive ou normative?  Participatory?  Empowering? Not guilt/blaming attitude?  Ability to switch from one attitude to the other? |  |
|  |  |
| Difficulties encountered  Conflict? Lack of pupils’ participation? Behaviour problem?  Students’ reaction(s)? |  |
|  |  |
| Class feedback  How do students obtain this feedback?  Other evidence of pupils’ understanding? |  |
|  |  |
